# Instantaneous center of rotation, the first step to build up the digital laboratory of complex motions

**DOI:** 10.1371/journal.pone.0329021

**Published:** 2025-08-07

**Authors:** Balazs Laczi, Antal Nagy, Arpad Safrany-Fark

**Affiliations:** 1 Department of Image Processing and Computer Graphics, Institute of Informatics, Faculty of Science and Informatics, University of Szeged, Szeged, Hungary; 2 Department of Oral and Maxillofacial Surgery, Albert Szent-Györgyi Medical School, University of Szeged, Szeged, Hungary; University of Perugia: Universita degli Studi di Perugia, ITALY

## Abstract

Calculating instantaneous centers of rotation to describe combined rotational and translational motions has a long history in many fields of applied science and basic rigid body kinematics. However, only some theoretical studies have explored the fundamental characteristics of this system. This study used digital three-dimensional modeling and computing methods to examine the system’s operation in a controlled in vitro-like environment. The effects of inaccurate registrations on the resulting motion were also analyzed. We registered 28.65, 14.33 and 9.55 EcD_ratios for 2°, 4° and 6° of closure respectively, and described a structured, predictable framework based on a solid mathematical background. Our findings align with previous publications, indicating that the longstanding debate over the pure rotation of the temporomandibular joint arises from misinterpretations of scientific findings due to a lack of fundamental knowledge of the basic characteristics of the system. Our simplified geometrical approach significantly reduces the complexity of the existing complex kinematic model, making it more accessible for practical applications, easier to understand, and potentially more applicable in orthopedics or temporomandibular joint radiology. We identified five fundamental characteristics of the system as we described the effects of the acting translational component in the complex motion. We also presented a detailed model concerning the effects of inaccurate rotation axis registration on the resulting compromised transformation, improving our understanding of the error tolerance level of articulation systems. Our results show that the system might tolerate errors as great as 3–4 cm in some settings in the parallel error direction, while in case of circular and perpendicular error types an approximately 2 mm axis registration error would exceed the clinically desirable 0.1 mm occlusal error level. Our experimental modeling strategy might provide extensive data for machine learning and for analyzing and comprehending the fundamental characteristics of different complex motion systems in the future.

## Introduction

Mandibular kinematics measurements are widely used for clinical purposes. Correlation between temporomandibular disorders (TMD) and mandibular kinematics has been well-documented in the literature [1–3]. Techniques for measuring mandibular kinematics can be categorized into four main groups: mechanical linkage systems, magnetic tracking systems, video motion analysis, and radiographic tracking [1,4]. Some of the key kinematic parameters are mandibular rotation, translation and “hinge axis” [1–3]. To facilitate calculations and evaluations performed on the data collected by these tools, it is essential to compile and summarize foundational knowledge related to Rotation Centers and Instantaneous Centers of Rotation (ICRs) in cases lacking pure rotation. A fundamental strategy to describe combined rotational and translational motion is to define it by its ICR. The easiest example of such a complex motion in our human world is a turning ship diverted from its course by sea currents. ICR describes the position of a point in a two-dimensional (2D) environment or an axis in a three-dimensional (3D) space that could theoretically function as a center of rotation and transfer the observed rigid body from its starting position to its final location during a given period (aka instant) solely with rotatory movement even though, in reality, the observed body might perform rotatory and translational movements simultaneously. Thus, the ICR describes the position of the center about which an object seems to be rotating at a given instant [5,6]. It is a strategy to describe a complex motion with a simplified method approximating the same result. Mathematically, it can be defined as the point in a 2D space or axis in a 3D space that maintains a constant distance from every point of the body or as the point with zero velocity during infinitesimally small motion [7].

ICR is used in many fields of applied science besides basic rigid body kinematics, such as aeronautics, radar imaging, robotics, descriptive kinematics of nautical and land transportation, and disciplines of medicine implying human joints [8–17]. Although the importance of a simplified approach to comprehending a complex problem cannot be underestimated, the extensive use of ICR in these fields has been awaiting since the 19^th^ century [18]. The primary reasons for the inextensive practical use of ICR and the lack of empirical results in the literature are its high sensitivity to noise and susceptibility to error magnification with small rotation angles [7,19,20]. While strategies, such as the symmetrical center of rotation estimation (SCoRE), sufficiently reduce these errors in ball joints where pure rotation can be assumed, in complex motions, they do not apply [21]. Noise sensitivity causes apparent randomness of ICR distribution during clinical investigations, which is highly unlikely in the natural environment, yet it prevents comprehension and limits the practical use of the method [22]. Unfortunately, only a few theoretical studies have been conducted to describe the basic characteristics of the system, of which the most notable is Mehl’s fundamental kinematic analysis on detecting the rotation axis of the Temporomandibular Joint (TMJ) with pantographic methods. His results show that only a small amount of transitional component during rotation significantly displaces the calculated ICR. A translational portion of only 1 mm displaces the center of rotation of around 6 mm, and in the 2 mm translation, the displacement is approximately 12 mm [23].

Our previous study concluded similarly, where we describe this “*transitional error-caused axis displacement ratio”* (EcD_ratio) in the 3D environment with a ratio of 41.83 to 1 (SD = 14.89); therefore, on average, a transition of 1 mm displaces the rotation axis by 41.83 mm. A higher rotation to the same amount of transition reduces this ratio, similar to Mehl’s results [23,24]. This can explain the noise sensitivity and the increased challenges in the case of small rotation angles. Another relevant study by Piehslinger et al. investigated the clinical effects of inaccurate registration of the rotation axis of the TMJ. They found that an axis shift of 5 mm causes an occlusal error greater than 0.1 mm [25]. This 5 to 0.1 ratio highly resembles our results, as we have examined the same coin from different sides [24,25]. While these findings explain the sensitivity to noise from a statistical descriptive viewpoint, they do not describe the seemingly random distribution and displacement of these ICRs during clinical trials.

We found that a digital in vitro study is needed for further understanding. We used the digital 3D space and different modeling and computing methods as an in vitro-like laboratory to examine the system operation in a controlled environment. In these circumstances, we can modify all aspects of the system separately; we can change the amount of the original rotation, the position of the original center of rotation, and the magnitude and direction of the transitional component that we add to the original rotation. Thus, we can describe the effects of these components on the position of the newly calculated ICRs.

We also examined the other aspects of the system in this controlled environment, primarily modifying the centers of rotation with known amounts and displacement directions, describing the effects on the resulting motion similarly to Piehslinger et al. [25]. We expected to find regularities that can be reported because we know that randomness in nature is highly unlikely, especially in straightforward systems like our subject of interest [22]. We also expected to find similar regularities when changing the variables affecting the ICR registration and by manipulating the center of rotation in the first place since Piehslinger et al.’s results highly resemble our previously published EcD_ratio [24,25]. In addition to the scientific relevance of such geometry, well-established findings could be an input for machine learning that might be used later in many applied fields of science. Our strategy to comprehend complex motion systems can be the first step to build more complex systems artificially with multiple rotational and translational components, e.g., simulations on planetary or projectile movements with more than one type of circular or rotational component and multiple diverting forces during the complex motion. Our aim was to inspect the two systems related to the ICR strategy of TMJ kinematics on a theoretical basis: The detailed effects of transitional components on ICR calculations (First experimental setup), and the possible effects of the registration errors of axes on clinical accuracy (Second experimental setup). We wish to present a detailed logical framework established in a noise-free highly controlled digital platform, which could help to evaluate clinical results of joint kinematics and other non-medical applications of ICR registrations.

## Materials and methods

Developing a system in a controlled environment where different variables can be modified artificially and independently is essential for in vitro investigations. While in vitro experiments in some fields, such as medicine, biology, or chemistry, typically require a complex laboratory background, in our field of interest, a digital 3D space might be a sufficient in vitro environment if equipped with adequate tools of 3D modeling and different computational methods free of material bounds.

### First experimental setup: Effects of the transitional components on the calculated ICRs in the sagittal plane

Our initial investigations aimed to describe any regularities that could be traced using fine-tuned ICR registrations performed on artificially positioned 3D meshes. These meshes were 3D scans of the lower dental arches captured during our previous clinical study [24]. The data acquisition of the previous publication and all related further investigations with artificial transformations were approved by the Human Investigation Review Board of Szeged University (approval no.: 43/2020-SZTE). The participants were recruited between 1^st^ of March and 31^st^ of April 2021. The STL files used for this study do not contain patient metadata and are fully anonymized. Two different scans were used with a clinically registered axis for each. The most centrally positioned axis was chosen from the clinical study and aligned to be parallel to the *x*-axis of our global coordinate system. The two meshes were used to control the effect of the mesh properties, such as size, shape, and the number of voxels, because it was expected to modify the final result of the ICR registrations significantly. The essential elements of our first experimental setup were the axis used for pure rotation and three identical but differently positioned meshes in the 3D space.

**Fig 1:**
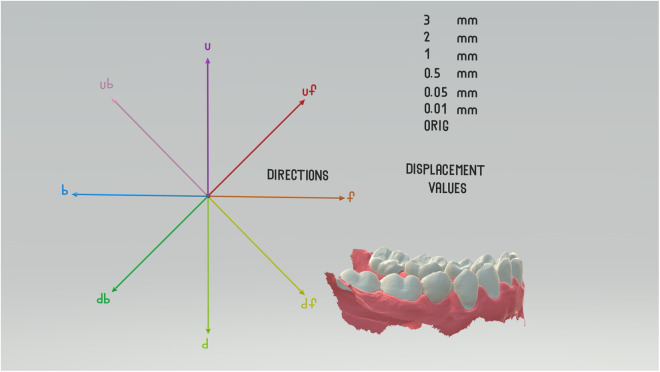
Schematic representation of the first experimental setup. A 2D version of the schematic 3D figure (S1 Fig, can be opened using Microsoft Paint 3D or other platforms handling.glb files). Please note: The original 3D files were excessively modified as they were painted manually to help identify anatomical parts such as teeth and gingiva, and to separate the *directions*. Relevant raw data and 3D files are available in the data repository, coordinates of the axes are included in the related Tables respectively.

The first was the original mesh (A_1_), the second was a mesh rotated by a predefined amount (*α*_1_) around the original axis of rotation (A_2_), and the third mesh was the target of two displacements: first, the same rotation (*α*_1_ around the original axis of rotation) and a linear shift by a predefined amount and direction simulating the transitional error (${\vec{t}}_{e}$) in our setup (A_3_). The shift was always perpendicular to the axis of rotation and, therefore, also perpendicular to the *x*-axis of the global coordinate system. The final element of our setup was a registration using the 3D variant of the Reuleaux method that produced the ICR, our target of interest [26]. The perpendicular plane on the *x*-axis can be interpreted as the theoretical sagittal plane of our digital patient. The variables in our setup were determined by ${\vec{t}}_{e}$ values with 0.01, 0.05, 0.5, 1, 2, and 3 mm respectively. The ${\vec{t}}_{e}$ values followed eight directions parallel to the sagittal plane with a 45° deviation from each other; four of them followed the primary directions of the *y*- and *z*-axes of the coordinate system, and four were set between them by 45°. The following abbreviations were used: b (back), d (down), db (down&back), df (down&forward), f (forward), u (up), ub (up&back), uf (up&forward) (Fig 1 and S1 Fig).

The *α*_1_ values were 2°, 2.5°, 3°, 3.5°, 4°, 4.5°, 6°, and 9°. All combinations of these variables were used for our registrations. Our primary output parameters were the 3D coordinates of our registered axis 100 mm from the closest point of the axis to the gravity point of the original mesh (A_1_), the distance of the original and registered axes (${\vec{d}}_{axis}$), the angular deviation of the original and registered axes, the angle of the registered transformation around the ICR (*α*_2_), the registration time (exec_time), and the rms error of the meshes after the transformation (rms in S1 Table). The most critical parameter derived was the EcD_ratio of each registration.

A preliminary overview of the results suggested that a universal geometrical phenomenon must rely on our findings. For further investigation, we extracted the coordinates of the same voxels of the three differently positioned (identical) meshes and the coordinates of the two axes (the original and calculated ICRs) on the intersection of the plane defined by the three identical points of the three meshes. We used GeoGebra (Byju’s, Bangalore, India) to reproduce the positions of our five points in 2D by their coordinates and search for the correct geometrical solution on this simplified platform. Thus, the problem was illustrated as a simplified 2D case of the 3D problem when the translation error vector is perpendicular to the rotation axes.

### Second experimental setup: Investigation of the effect of registration errors of the axes on the position of the rotated objects

**Fig 2:**
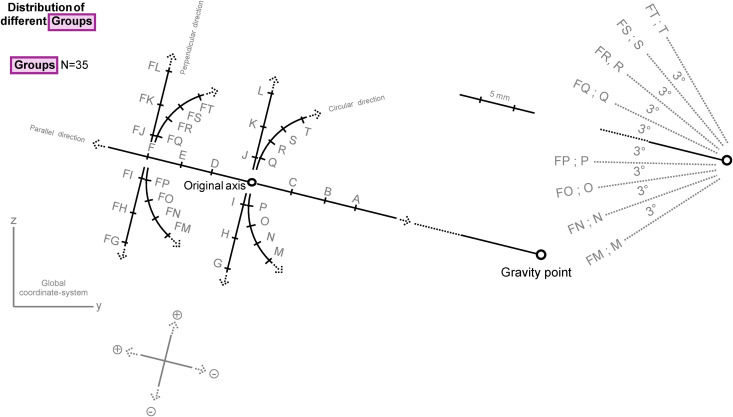
Schematic representation of the distribution of different “groups” of second experimental setup.

**Fig 3:**
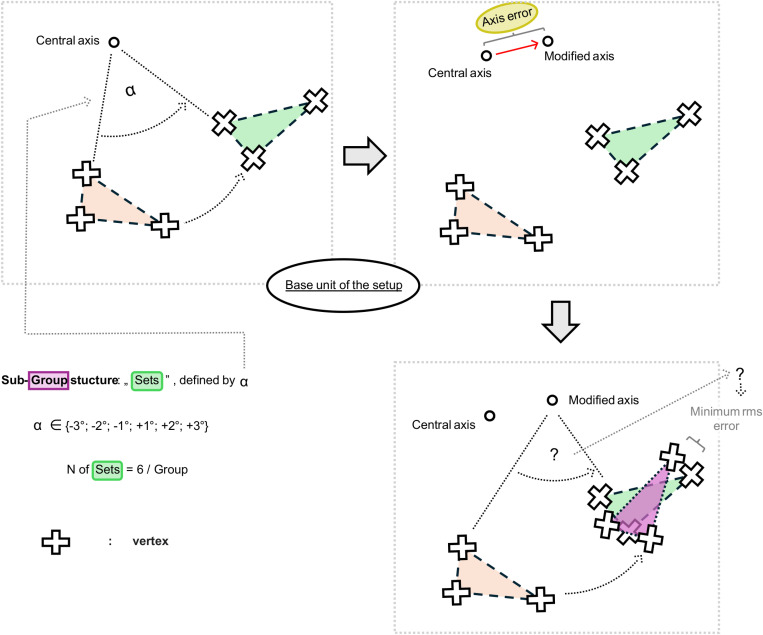
Schematic representation of the logical background of the “sets” of second experimental setup.

**Fig 4:**
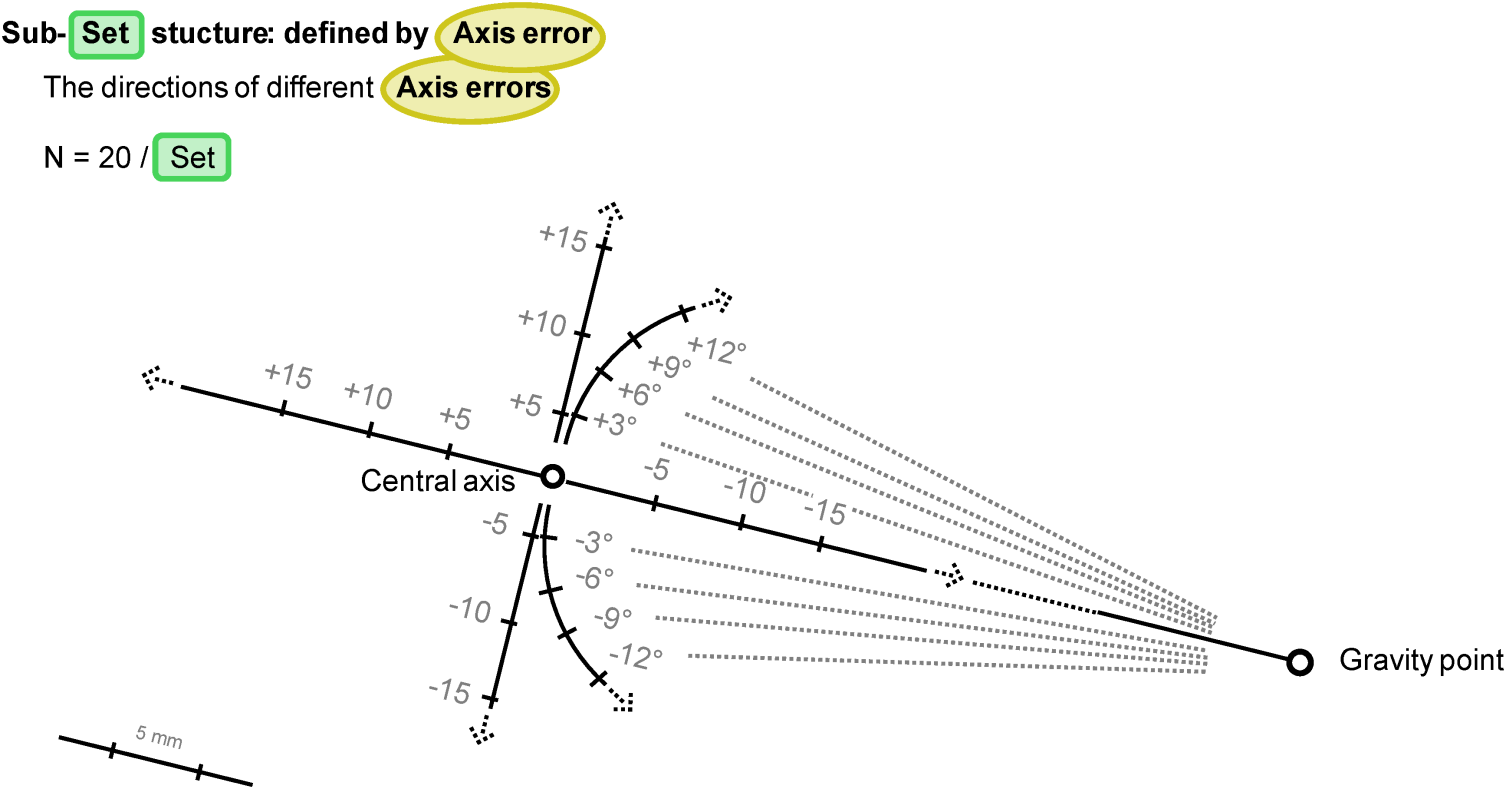
Schematic representation of the orientation of the specific errors used in the second experimental setup.

We analyzed the effects of the registration errors of the rotation axes by creating a series of artificially misplaced axes. The in vitro-like digital setup allowed us to add predefined errors (by type and magnitude) and describe the consequences of such errors on the resulting transformations. The base unit of our setup contains five elements: an unmodified central axis and an artificially misplaced axis representing the hypothetical error of the axis registration. It also includes an initial, unmanipulated mesh that was the target of two transformations (both pure rotations): a rotation around the unmodified central axis by a predefined amount of −3°, −2°, −1°, + 1°, + 2°, and +3° (+ and − represents the rotation directions; Figs 2–4), and another rotation around the artificially modified axis until the best possible overlap is reached (defined by the lowest possible rms_value) with the previously rotated mesh. Our previously published optimizer method was used for this rotation in a simplified manner [24]. This setup represents a correct transformation by the accurately registered axis and an incorrect transformation by an inaccurate axis (Fig 3). Multiple unmodified central axes were used at different positions in the 3D space to control the effect of the prime location of the axes on the results, each defining a “Group” of axes derived from it (Fig 2). A specific amount of rotation of the six rotation values defines a “Set,” a specific subgroup level (Figs 3, 4).

Three modifications were performed on the unmodified central axes: linear shifts along the connecting line between the closest point of the central axis and the gravity point of the initial unmanipulated mesh by −15, −10, −5, + 5, + 10, and +15 mm (+ and − represent the displacement directions, where − is the direction toward the mesh, this line of movement is called the parallel path or parallel direction), linear shifts perpendicular to the unmodified central axis and the parallel path by the same amounts (the + direction is closer to the z + direction of the global coordinate system), and rotations around the gravity point of the initial mesh parallel to the central axis by −12°, −9°, −6°, −3°, + 3°, + 6°, + 9°, and +12° (+ and − represent the rotation directions closer to the analog directions of the perpendicular path). These 20 modified axes with the same initial mesh rotation value make up the “set” of previously mentioned axes.

All six sets of different rotation values and the unmodified central axis make up the group as a unit. The group positions were chosen carefully to be sufficient to investigate the effect of the distance and location of the central axis from the meshes (Fig 2). Furthermore, we created two experimental setups with the same pattern. For these two setups, we used two meshes of two scans of the lower dental arches of our previous clinical study, but the same clinically registered axis was used for both meshes [24]. The gravity points of the scans were matched (the relation of the axis to the unaligned mesh was kept at this step, but then it was aligned to be parallel to the *x*-axis of the global coordinate system). This first axis was named the “original axis” and functioned as the first unmodified central axis of the first group. Six groups were created parallel and six perpendicular from the original axis (Fig 2). Eight groups were created in a circular path around the gravity points of the meshes, similarly to the in-group error creation (Fig 2). Each group was named by letters A–T. The farthest parallel central axis (F) was used to create an additional series of perpendicularly and circularly positioned groups (the FG–FT groups) to control the effect of the mesh to central axis distance on the results regarding the perpendicular and circular directions. The two meshes were used to control the effect of the mesh properties on our results.

We described the primary characteristics of the system by calculating the error ratio of the artificially created axes (the distance of the unmodified central and artificially shifted/rotated axes in mm) and the rms errors of the rotated meshes (one rotated by the unmodified central axis and the other rotated by the artificially modified axis) for each pair. This value can be found under the Axis error-caused Final error (AEcFE) in the S2 Table. The easiest way to comprehend the AEcFE values is if we interpret these values as the amount of error (mm) that would cause a 1 mm rms_error of the meshes. Thus, a higher value means less sensitivity to the error type or direction.

An in-depth investigation of the results at the subgroup level can provide information on different error directions and their effects on the final rms error of the meshes. However, intergroup bulk statistics were also needed to make such a considerable amount of data comprehensible.

For statistical analysis (of both Setups) we grouped the data by rotation angles. Wilcoxon Signed Rank Tests were used for related samples or one sample cases. For independent-samples cases we used Kruskal-Wallis Tests. All evaluations were conducted in IBM SPSS Statistics for Windows, Version 27.0. Armonk, NY: IBM Corp.

## Results

### Effects of the transitional components on the calculated ICRs in the sagittal plane

Manipulating simple 3D objects, such as intraoral scans (digital impressions of dental arches), revealed that we highly underestimated the possible regularity of the distribution of the calculated axes (ICRs in the 3D space) if only a single transitional component affects the rotation at a time. We could determine some regularities by merely observing the axes (available in the data repository). The axes belonging to the same amount of initial rotation but different amounts of ${\vec{t}}_{e}$, which were added to the system to modify those original meshes and the registered axes, are always distributed by following rules. The EcD_ratio belonging to a particular angle of initial rotation remained the same regardless of the amount of the ${\vec{t}}_{e}$ and the position of the original axis (${\vec{t}}_{e}$ *is always perpendicular to the rotation axes; for more detail, see* *Materials and Methods* *or later sections related to our 2D analytical calculation*). The calculated axes are always oriented following a rule that we can describe as 90° − *α*_1_/2 away from the original axis, where *α*_1_ is the angle of the initial rotation. The axes of the same *α*_1_ with different amounts of ${\vec{t}}_{e}$ added to the system are lined up following a plane oriented from the initial axis in the direction of 90° − *α*_1_/2 away from the direction of the$\;{\vec{t}}_{e}$, with the larger amount of ${\vec{t}}_{e}$ positioned further. The 90° − *α*_1_/2 rule of the distribution of the axes was the most comprehensible finding by mere visual inspection of our results. However, further observations were also made which might be relevant for practical uses. We can summarize (*with a highly oversimplified, yet more comprehensible manner*) the following characteristics of the ICR system where the translation vector is perpendicular to the rotation axes (in these major findings there were no practical differences between the rotation angle groups):

1) The new rotation angle around the ICR practically equals (with only minor numerical differences on a 10^−6^ scale in degrees) the original rotation angle: *α*_2_ = *α*_1_.
2) The ratio of the amount of translational component and the shift of the calculated ICR from the original center of rotation (EcD_ratio) is practically constant for each different rotation angle.
3) The direction of the axis shift is predictable if the direction of the translational component and the original rotation angle are known (90° − *α*_1_/2).
4) These findings are universal in space and seemingly independent of the properties of the observed body. Thus, in our experimental setup, the distance of the axes from the mesh and the mesh properties did not change these system characteristics.
5) The substitutional rotation around the ICR transfers the mesh almost to the exact location where the combined motion would position it (final rms error values on a 10^−7^ mm scale); thus, the calculated transformation does not approximate but practically substitutes the original one in the sense of the final position, despite that the path of movement is not the same.

The detailed presentation of these results and the related statistics can be found in S1 Text and S1 Table.

**Fig 5:**
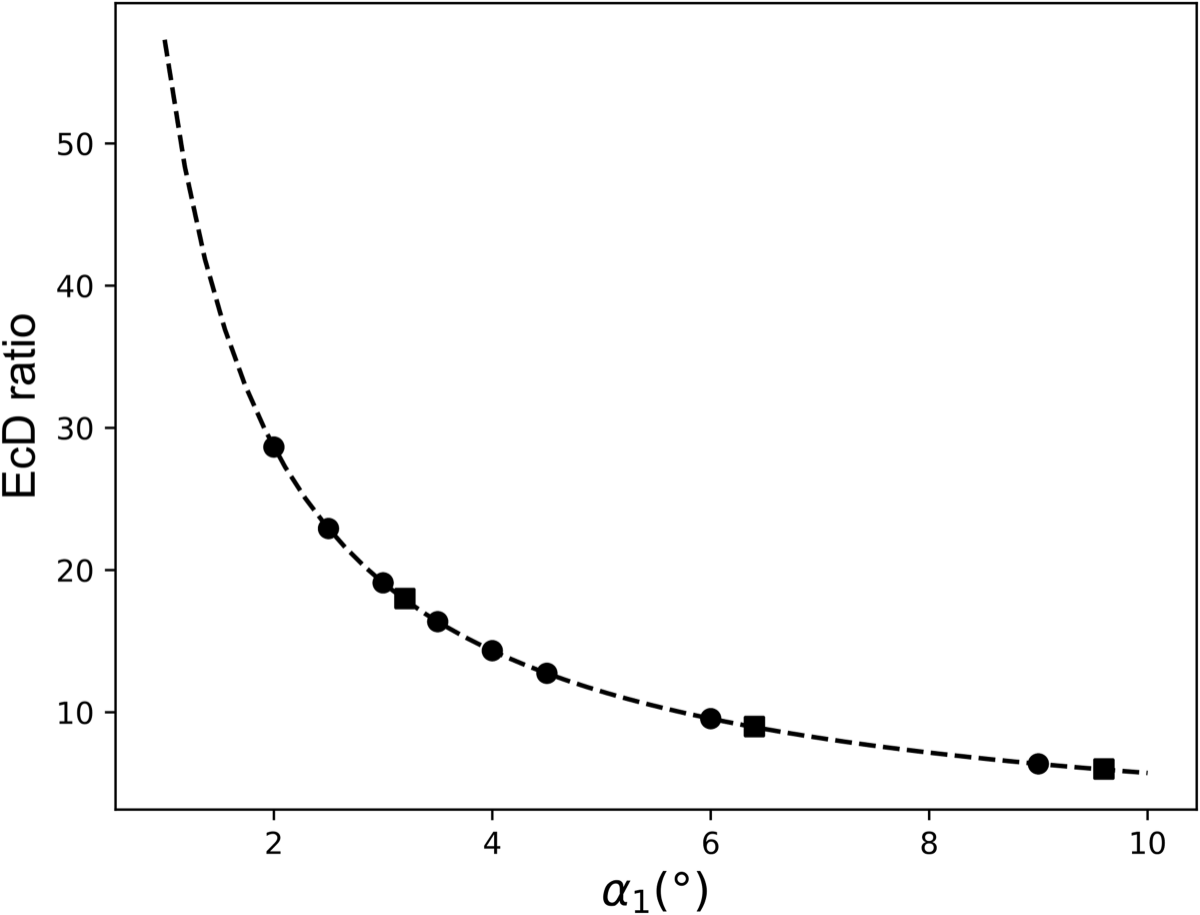
EcD_ratios of different degrees of closure. Data shown by dots represent our results, whereas the squares represent the EcD_ratios derived from Mehl’s published results [23].


The EcD_ratios of different *α*_1_ values followed a function (Fig 5):

$$EcD=\frac{57.2880981}{{\alpha_{1}}^{0.999893022}}+0.00303367017.$$

The EcD_ratios derived from Mehl’s results fit well on our curve, proving a universal finding because the two experimental setups cannot be further methodically (Fig 5; S1 Table; Table 1).

**Table 1:** Data used for Fig 5. EcD_ratios of different degrees of closure.

	α_1_ (°)	EcD_ratio	curve_fitting_value	Difference		
MESH I	2.00	28.65	28.650965	0.000965		
	2.50	22.92	22.926207	0.006207		
	3.00	19.10	19.109273	0.009273		
	3.50	16.37	16.382637	0.012637		
	4.00	14.33	14.337498	0.007498		
	4.50	12.74	12.746724	0.006724		
	6.00	9.55	9.564831	0.014831		
	9.00	6.37	6.382343	0.012343		
MESH II	2.00	28.65	28.650965	0.000965		
	2.50	22.92	22.926207	0.006207		
	3.00	19.10	19.109273	0.009273		
	3.50	16.37	16.382637	0.012637		
	4.00	14.33	14.337498	0.007498		
	4.50	12.74	12.746724	0.006724		
	6.00	9.55	9.564831	0.014831		
	9.00	6.37	6.382343	0.012343		
	Absolut mean error: 0.001991					
					Translation	d_axis_
Mehl A.	3.20	18.00	17.916398	0.083602	0.50	9.00
	3.20	18.00	17.916398	0.083602	1.00	18.00
	3.20	17.99	17.916398	0.076935	1.50	26.99
	3.20	17.99	17.916398	0.073602	2.00	35.98
	6.40	9.00	8.968166	0.031834	0.50	4.50
	6.40	8.98	8.968166	0.011834	1.00	8.98
	6.40	9.00	8.968166	0.031834	1.50	13.50
	6.40	9.00	8.968166	0.031834	2.00	18.00
	9.60	6.02	5.984477	0.035523	0.50	3.01
	9.60	6.00	5.984477	0.015523	1.00	6.00
	9.60	6.00	5.984477	0.015523	1.50	9.00
	9.60	6.01	5.984477	0.020523	2.00	12.01
	Absolut mean error: 0.053742					

Our results compared to those derived from Mehl’s publication [23]. Original datasets compared to the values of the proposed curve. The difference in these values is informative for judging the fitness of the original data to the curve. “Translation” and “daxis” values are Mehl’s original data.

The two most critical findings at this point were the following:

1. The calculated errors of the different metrics were confusingly low, almost as if they only resulted from computational errors.
2. We found that only by observing a simple 3D in vitro setup we can predict the effects of the different components of our system with an enormously high probability.

**Fig 6:**
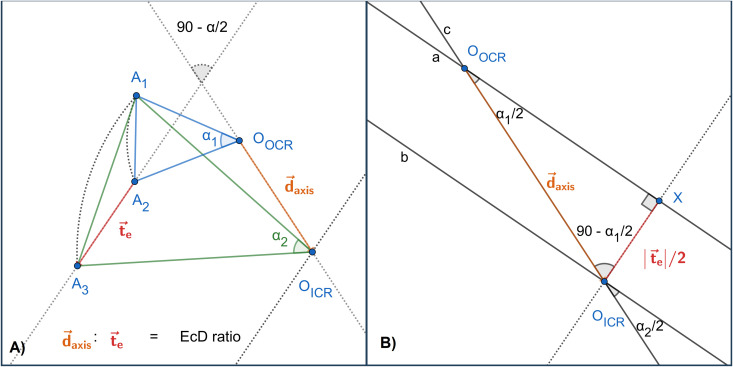
The simplified 2D representation of the experimental setup of the ICRs (A) and the related analytical calculation (B). Fig 6A shows the original complex transformation (the rotation around the O_OCR_ and the translation of the ${\vec{t}}_{e}$ vector) and the simplified resulting transformation (the rotation that only used the O_ICR_ as the rotation center point) through the different stages (A_1_–A_3_) of point A (I: target mesh (A_1_), II: purely rotated mesh by *α*_1_ (A_2_), and III: the mesh rotated by *α*_1_ shifted by ${\vec{t}}_{e}$ (A_3_); mesh III was rotated back along the calculated ICR axes by the calculated *α*_2_; see Fig 6A). It is a plan view (2D) of the 3D system where the center of the rotation points represents the center of the rotation axes. Fig 6B shows that the rotation and translation are constructed by reflections using the *a*, *b*, and *c* mirror axes.

Here, we present a mathematical solution for the 2D representation (Fig 6) of the original 3D problem. However, it is critical that our solutions can be transformed back to the original 3D system by the same transformation used in [26]. We will show the analytical calculation to determine the new O_*ICR*_ position from the original O_*OCR*_ center of rotation. Finally, we give a vector ${\vec{d}}_{axis}\;$ as a function of the rotation and ${\vec{t}}_{e}$ translation.

Let us define:

- ***R***(*α*, O) as a rotation around the O point by *α* degree
- ***M***(*s*) as a reflection transformation through the mirror axis (line) *s*
- ***T***$\left(\vec{t}\right)$ as a translation using the $\vec{t}$ vector
- ***I*** as an identity transformation

First, we prove that ${T\left({\vec{t}}_{e}\right)R}_{1}\left(\alpha_{1},\;O_{OCR}\right)=R_{2}\left(\alpha_{2},\;O_{ICR}\right)$, where *α*_1_ = *α*_2_, meaning that the combination of the ***R***_1_ rotation with *α*_1_ around the O_*OCR*_ center and the translation with the ${\vec{t}}_{e}$ vector, we can achieve with a single rotation around the new O_*ICR*_ with the same angle. The original proof can be found in Pogáts [27]. For clarity, we summarize the most critical steps.

Rotation and translation can be produced by two reflections through the mirror axes [28].

For translation, the distance between the two mirror axes is half the length of the translation vector (in our case, $\left|{\vec{t}}_{e}\right|$/2), and the mirror axes are perpendicular to the translation direction. We also mention that the only condition here is the distance between the two parallel mirror axes. The first axis can be placed anywhere in the 2D space. The second axis should be in the translation direction.

For rotation, the angle between the two mirror axes is equal to half the rotation angle (in our case, *α*_1_/2), and their intersection point should be in the center of the rotation, in our case O_*OCR*_. The axis positions can be anywhere in the 2D space if they go through O_*OCR*_ and the angle condition is satisfied.

To create the composition of the rotation and translation transformations, let us assume that the ***R***_1_ rotation and the $T({\vec{t}}_{e})$ translation share a mirror axis *a* that goes through O_OCR_. Thus, ***R***_1_(*α*_1_, O_*OCR*_) can be expressed as a reflection of ***M***(*a*)***M***(*c*), where *c* is the first mirror axis and *a* is the second mirror axis, and the angle between the two mirror axes is *α*_1_/2. Also, the $T({\vec{t}}_{e})$ translation can be described as ***M***(*b*)***M***(*a*).

According to our setup, where mirror axis *a* is perpendicular to the direction of the ${\vec{t}}_{e}$ translation vector (Fig 6) and the ***M***(*a*)***M***(*c*) = ***I***, the given transformation can be written as


$$\begin{array}{c} {T\left(\vec{t_{e}}\right)R}_{1}\left(\alpha_{1},\;O_{OCR}\right)=\;\left(M(b)M(a)\right)\left(M(a)M(c)\right)=\;\\ =M(b)\left(M(a)M(a)\right)M(c)=\;M(b)IM(c)=M(b)M(c). \end{array}$$

The mirror axes *a* and *b* are parallel, and the angle between the *b* and *c* mirror axes is *α*_1_/2, thus, *α*_2_ = *α*_1_, which gives


$${T\left({\vec{t}}_{e}\right)R}_{1}\left(\alpha_{1},\;O_{OCR}\right)=M(b)M(c)=R_{2}\left(\alpha_{1},\;O_{ICR}\right).$$

Using this property, we can express the new position of the O_*ICR*_ center of rotation. Let *X* be the intersection point of the line perpendicular to line *a* and go through O_*ICR*_, the new center of rotation. The O_*ICR*_O_*OCR*_*X*_∆_ triangle is a right triangle according to our transformation setup. Applying the definition of the translation $T\left({\vec{t}}_{e}\right)$, we know that the length |O_*ICR*_*X*| equals the half-length of the ${\vec{t}}_{e}$ vector. Additionally, using the definition of the ***R***_1_ and ***R***_2_ rotations, the other two angles of the O_*ICR*_O_*OCR*_*X*_∆_ right triangle can also be determined. Based on these facts, we can determine the length of the ${\vec{d}}_{axis}\;$as follows, using the sine law,


$$\left|{\vec{d}}_{axis}\;\right|=\frac{\left|{\vec{t}}_{e}\right|}{2\;\sin\left(\frac{\alpha_{1}}{2}\right)}$$

where $\sin\left(\frac{\alpha_{1}}{2}\right)\neq 0$, meaning that *α*_1_ ≠ *k* * 360° (k∈ Z). The rotation with angle *k* * 360° is the identity transform. In this case, the new transformation does not contain any rotation.

When α_1_ = 180°, the O_*ICR*_ and *X* points are in the same location. In that case, $\left|{\vec{d}}_{axis}\;\right|=\frac{\left|{\vec{t}}_{e}\right|}{2}$.

The direction of the ${\vec{d}}_{axis}$ vector can be interpreted relative to the ${\vec{t}}_{e}$ translation vector, quantified in the same rotation direction as the original ***R***_1_ rotation. The angle between the ${\vec{d}}_{axis}$ and ${\vec{t}}_{e}$ vectors equals $90^{\circ }-\frac{\alpha_{1}}{2}$. The rotation angle should always be in the [0° − 180°] interval. If the *α*_1_ rotation angle is greater than 180°, it can be interpreted as a rotation in the opposite direction with the angle of 360° − *α*_1_.

The ${\vec{d}}_{axis}$ vector is oriented toward the ${\vec{t}}_{e}$ translation vector for the unique case when *α* = 180°.

The length and direction of the ${\vec{d}}_{axis}$ vector can be determined using the function of the ${\vec{t}}_{e}$ vector and the *α*_1_ rotation angle; thus, the location of the new rotation axis can be calculated.

Our simplified experimental setup can be built up in GeoGebra by anyone who seeks to reproduce our findings (an example is provided in the S1 File GeoGebra Project).

### Investigation of the effect of registration errors of the axes on the position of the rotated objects

We found higher mean AEcFE values on the parallel groups, which gradually increases as they move further from the gravity points of the meshes. Thus, the same amount of error has less effect on the mesh position from a further direction.

The perpendicular error-type mean AEcFE values of the same group are much smaller than the parallel type with the same amount of error; thus, this direction is more sensitive to the error. However, these AEcFE values remain stable in the intergroup statistics, even if the central axes are distributed on a parallel path. The mean AEcFE values of the perpendicular error type decrease slightly on the parallel path (less than 1%), whereas the circular error-type changes only to a negligible extent (on the 0.01% scale). All these mean AEcFE values remain stable in the intergroup comparison on the perpendicular path, with the highest change in the parallel error type (1.4%–2%), slightly smaller on the further path originating from Group F. There is no change in these values on the circular path between the groups. We could interpret these findings as circular zones around the meshes which produce a similar sensitivity to the registration errors. Parallel errors have the smallest effect on the mesh position, gradually decreasing further from the mesh, whereas errors in the perpendicular direction are more clinically dangerous.

Despite the dataset being grouped by the rotation angles we have not found fundamental differences in the test results between these groups. We found significant difference between the rms_after values of the two meshes if all error types were treated as single-grouped data and divided by the error types for all angle groups. These differences can be explained as consequences of unique meshes properties (shape, complexity, resolution, number of points), as in case of this Setup the performed transformations do not substitute the original rotation (contrary to the case of the First experimental setup), and the rms error values are calculated using the vertex points of the meshes, thus basic properties like the number of points in the meshes, have fundamental effects on the analysis. Thus, in the case of failed axis registrations, our findings can be interpreted as general trends characterizing the effects of unsuccessful registrations rather than strict numerical conclusions, contrary to the findings regarding ICR registrations where mesh properties do not change the registration results similarly.

**Fig 7:**
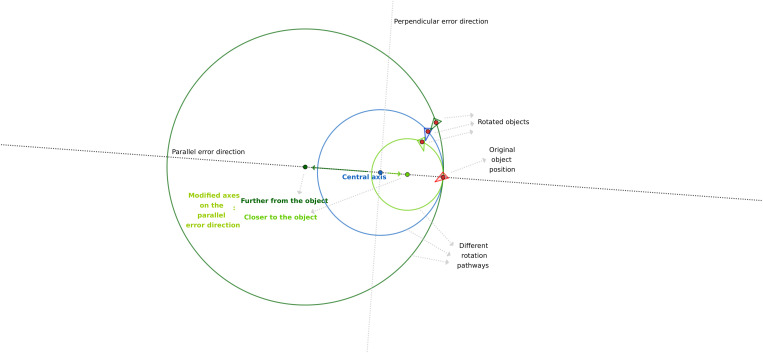
Schematic explanation of the effect of the registration errors in the parallel direction on the position of the rotated objects.

**Fig 8:**
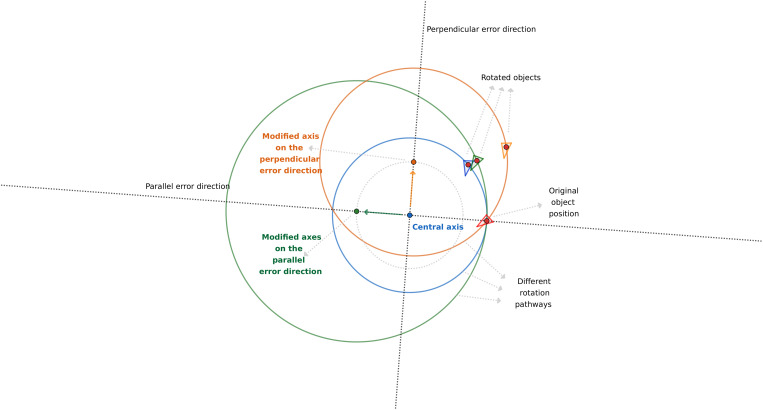
Schematic explanation of the different effects of registration errors in the parallel and perpendicular directions on the position of the rotated objects.

**Fig 9:**
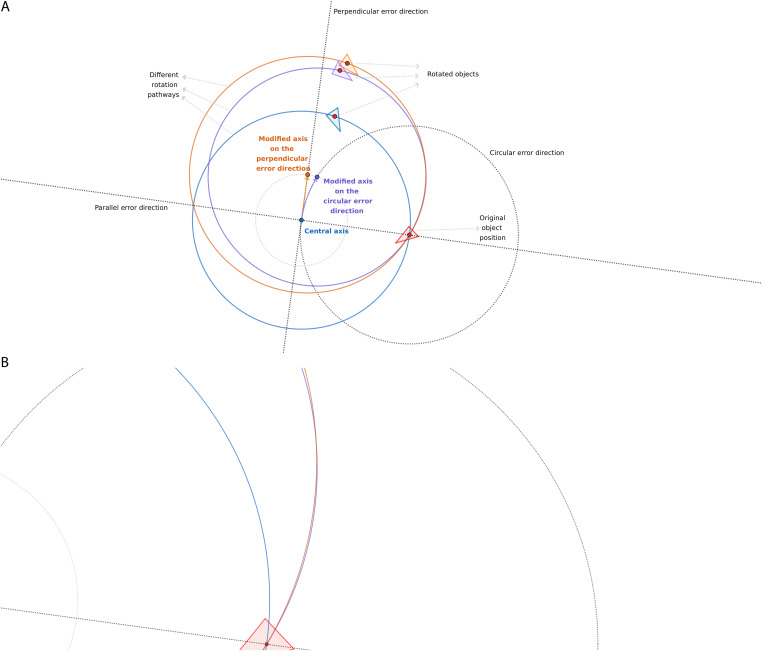
Schematic explanation of the different effects of registration errors in the circular and perpendicular directions on the position of the rotated objects. (A) The general relation of the two pathways. (B) The intersection of the two paths close to the mesh in a specific combination of variables.

We found that using deductive figures (designed in GeoGebra, Byju’s, Bangalore, India) is the easiest method to comprehend these results. These figures visualize the rotational pathways of different error types and the relationships of these pathways to each other (Figs 7–9).

In the parallel error direction, the circular pathway of a given rigid body follows an enlarged circle because the registration error modifies the original center of rotation further from the mesh, whereas it follows a constricted circle if the error acts toward it (Fig 7). In the case of the perpendicular error type (compared to the parallel one in Fig 8), the whole circle representing the pathway moves bodily sideways, causing an increased scissors-like opening of its arc from the original errorless (blue) path. The perpendicular and circular error types follow much closer paths; however, their relationship is more complex (Fig 9A). The circular error type produces a smaller circle for the same amount of error, which is logical because it is closer to the mesh. On one side of the pathway, the perpendicular, on the other side, the circular, situates closer to the original (blue) path. However, this is not always true because the two paths intersect close to the mesh in some specific combinations of the variables (Fig 9B).

Detailed examination of the results shown in the S2 Text might be time-consuming. The interpretation of these data shows that the least-tolerant AEcFE value of the circular error type (19.2 mean value) indicates that an approximately 2 mm axis registration error would exceed the clinically acceptable 0.1 mm error level, whereas parallel errors would cause at least four times less error on the level of the rotated scans [25]. The system might tolerate errors as great as 3–4 cm in some settings. These experimental results are hardly applicable in clinical situations, yet they describe some fundamental properties of the system. These findings might help structure clinical trials on the topic in the future.

## Discussion

First, we examined the fundamental logical background behind the ICR theory and summarized our observations in five main points. Most of these findings are geometrically predefined and are in agreement with previous publications [27]. Although Chen presented the clinical limitations of ICR calculations, it remains an essential investigative tool because it is the most profound approach to managing complex joint motions [25]. Mehl established a solid ground for understanding error sensitivity based on his fundamental kinetics. Although we used a different mathematical approach, it is crucial to state that it is merely an alternative to the previously published derivation of Mehl’s [23]. For detailed discussion of the relation of the presented mathematical background to previous publications and the questions of novelty see: S3 Text. We also presented our results for further visualization as freely importable 3D objects to any open-source computer-aided design software in our data repository. The 3D display and the figures in this paper might help deeper comprehension of the topic for the border clinical and scientific community not specifically involved in similar investigations but still affected by the limitations of and seemingly abstract characteristics of the discussed systems [29]. For the same reasons, we paid particular attention to comprehensible metrics, such as the EcD_ratio and AEcFE values, reviewed previously published information, and compared our findings. We discussed the basic characteristics of these systems more practically and supplemented them where missing information was found. Our simplified approach should reduce the complexity, making the solution more accessible for practical applications, easier to understand, and potentially more applicable in fields such as orthopedics or TMJ radiology (real-time TMJ MRI investigations and registration of the center of rotation of ball joints) [30,31]. By refining existing approaches, our mathematical solution might contribute to incremental advancements that facilitate broader use and inspire further research on related topics.

Furthermore, we must determine whether these errors act in the same way with clinical tools designed to reproduce patient-specific motion. Do they compromise the outcomes of different clinical workflows based on intraoral scanners, virtual articulators and motion-tracking devices? Digital impression techniques are subjects of rapid evolution [32]. However, some concerning results have been reported on bite alignment accuracy [33–36], similarly to our previous findings [24]. The possible impact of our findings on these clinical tools are discussed in S4 Text.

Second, we examined the effect of inaccurate rotation axis registration on the resulting compromised transformation. Previously, we found similar registration-error-caused effect ratios to Piehslinger et al.’s [24,25]. We expected to find similarities in the basic logic of the two systems. The observations defied all these presumptions. The fundamental logic regarding the error-caused effect of the second observed system can be summarized as highly tolerant to articulating inaccuracy and dependent on the distance and direction of the error and the properties of the observed body. Our results correlate with previously published findings [25,37–39]. However, most investigations chose a different approach to the problem or focused solely on one aspect of the presented model, which we called the parallel error direction, and thus failed to describe the system as a whole and clarify its logical background. Furthermore, the least-tolerant aspect of the model remained unknown during these investigations because none of these papers discussed the circular error type or the circular orientation of the results.

These summarized characteristics of both systems related to our previously proposed scanner-based methodology for rotation axis registration shed new light on the potential of such a tool [24]. The distribution range of the calculated ICRs was comparable in magnitude to the relation of the kinematically and conventionally defined arbitrary axes of the facebow [25,40,41]. Further improvement of bite alignment accuracy and optimization of the clinical protocol could reduce the registration range below the desired maximum of 3–5 mm. Both liabilities are subjects of current interest in the field [33,42,43]. Jaw-tracking systems, such as Modjaw, can validate clinical protocols applied with our scanner-based methodology because both are based on digital platforms.

## Conclusions

The most tangible conclusion of our study concerns the decade-long scientific dilemma of the true hinge axis versus ICR theories of the TMJ, i.e., is there a pure rotation stage of the joint during the initial opening stage? During these decades, the scientific community missed a more fundamental question: Do we have adequate methods to make registrations sensitive enough even to start a discussion? Frequently used pantographic methods are likely insufficient for such investigations, as stated by Mehl [23]. A lack of basic knowledge about the system applied during these clinical investigations led to premature conclusions. First, fundamental research must be performed to understand the characteristics of the applied system. In our case, we must understand that only a minor transitional component, naturally present or a result of registration errors, significantly modifies the position of the calculated ICR. The magnitude of technological and clinical accuracy required makes applying any registration tool based on calculating ICRs challenging. Thus, we cannot rely on these methods during theoretical discussions on human joints. Despite the challenges, ICR registration might have a relevant place in the clinical toolset if the outcome meets the clinically required level of precision, according to Piehslinger et al.’s argument on the topic [25].

The next logical step of investigation is to add factors such as time and velocity to the system, which will significantly increase the complexity of the mathematical background. However, it might still be comprehensible by systematic modeling and analysis. Building relevant artificial frameworks gradually and using similar in vitro-like digital setups could be a valuable input for machine learning. Thus, experimental modeling might have more relevance in our machine-learning-fueled world than ever before. However, this platform has a considerable disadvantage that must be considered. Despite the model we described in our mathematical argument where ICRs work errorless in 2D, our registrations produced numerically minor but still present rms errors in the 3D setup, although these errors should be zero if mimicking the 2D findings. Most likely, it was caused by rounding or computing errors that are naturally present in the computational system. With more complex motions, these errors might be serious obstacles produced by this framework and must be addressed.

## Supporting information

S1 TableThe primary results of the registered ICRs of our first experimental setup.Column “E” labeled “Case” contains the *α*_1_ value, the direction and magnitude of the $\vec{t_{e}}$ applied on the mesh, in that order. Column “W” labeled “angle_important” contains the registered value identified as (90° − *α*_1_/2) during the first inspection of the registered and 3D displayed data. Obj. files can be used for inspection and are available for the data repository. In Obj. files oriented “Forward: -Z & Up: In Y,” some software mixes these orientations during default import. The blender should be set to Y Forward and Z Up. The coordinates of the registered ICRs are also included at the end of the table.(XLSX)

S2 Table The primary results of the second experimental setup designed to investigate the effects of registration errors. Two sheets were used for each mesh. The first contains the registered data and derived values, and the second is used for bulk statistics.
(XLSX)

S1 File GeoGebra Project. The 2D simplified experimental setup for the ICRs. The setup was built up by two points rotated by the same amount on different circular paths because they would be two points of the same rigid body. After the rotation, both points shifted on the parallel lines by the same amount, representing the translational component. The Reuleaux method (purple lines in the presented S1 File GeoGebra Project) can be used to find the ICR. After manipulating the original values, the correctly constructed set maintains equal translation and rotation values. Our findings can be empirically verified using this straightforward tool.
(ZIP)

S1 Fig Schematic representation of the first experimental setup. Openable in a simple 3D paint tool or other platforms handling.glb files. Please note: The original 3D files were excessively modified as they were painted manually to help identify anatomical parts such as teeth and gingiva, and to separate the directions. Relevant raw data and 3D files are available in the data repository, co-ordinates of the axes are included in the related Tables respectively.
(GLB)

S1 Text In depth presentation of results of the First experimental setup. (DOCX)

S2 Text In depth presentation of results of the Second experimental setup. (DOCX)

S3 Text Statement of novelty and relation of the presented mathematical background to previous publications. (DOCX)

S4 Text The detailed discussion of the issue of error sensitivity on the clinical tools of digital dentistry. (DOCX)
