# Supplementary material for: Instantaneous center of rotation, the first step to build up the digital laboratory of complex motions
Source: PLoS One. 2025 Aug 7;20(8):e0329021. doi: 10.1371/journal.pone.0329021 (PMC12331127; doi:10.1371/journal.pone.0329021)
Supplement: S1 Text — (DOCX) [file pone.0329021.s005.docx]

**The detailed presentation of these results and the related statistics of the first experimental setup: Effects of the transitional components on the calculated ICRs in the sagittal plane**

Note: A summary table of the *p* values are attached to the end of this document, statistical analysis reports are also available in the data repository

Manipulating simple 3D objects, such as intraoral scans (digital impressions of dental arches), revealed that we highly underestimated the possible regularity of the distribution of the calculated axes (ICRs in the 3D space) if only a single transitional component affects the rotation at a time. We could determine some regularities by merely observing the axes (3D models available in the data repository). The axes belonging to the same amount of initial rotation but different amounts of $\vec{t}_{e}$, which were added to the system to modify those original meshes and the registered axes, are always distributed by following rules. The EcD_ratio belonging to a particular angle of initial rotation remained the same regardless of the amount of the $\vec{t}_{e}$ and the position of the original axis ($\vec{t}_{e}$ *is always perpendicular to the rotation axes; for more detail, see Materials and Methods or later sections related to our 2D analytical calculation*). The calculated axes are always oriented following a rule that we can describe as 90° − *α*_1_/2 away from the original axis, where *α*_1_ is the angle of the initial rotation. The axes of the same *α*_1_ with different amounts of $\vec{t}_{e}$ added to the system are lined up following a plane oriented from the initial axis in the direction of 90° − *α*_1_/2 away from the direction of the$\vec{t}_{e}$, with the larger amount of $\vec{t}_{e}$ positioned further. The 90° − *α*_1_/2 rule of the distribution of the axes was the most comprehensible finding by mere visual inspection of our results. The expected and registered angle values are closely correlated (the absolute difference in degrees: mean = 0.00115696, SD = 0.0027941; S1 Table). The two meshes of the two participants (two meshes were used to control the effect of mesh properties on our results: Mesh I and Mesh II, see Materials and Methods) provided similar registered values (the difference proved to be statistically insignificant for all of the rotation angle groups; for more details see the statistical analysis reports available in the data repository or in the summary table), indicating that a mesh-independent system relied on these registration results, which was confirmed later.

The artificially calculated transformation, which is the rotation (α2) along the calculated ICR axis, almost equaled α1 at all times (the absolute difference calculated for all rotation groups, in degrees: mean = 0.00000114, SD = 0.0000011). Similarly to the previous results, the calculated transformations (α2) for the two meshes of the two participants provided nearly equal values. However, this difference between the angles (*α*_2_) related to different meshes was statistically shown to be significantly different (*p* values can be found in the statistical reports available in the data repository or in the summary table), in the context of practical use these differences are so small (for all of the angle groups the absolute mean and the standard deviation values are in the 10^-6 scale or less, calculated in degree) that can be considered to be negligible. The final rms error of the meshes was confusingly low (calculated for all rotation groups: mean = 0.00000093 mm, SD = 0.00000044). This value describes the overlap of the mesh rotated by the ICR axis calculated by the 3D variant of the Reuleaux method by the amount of new rotation (*α*_2_) with the target mesh; thus, this value represents the correctness of the transformation [1]. However, this rms value also differed significantly between the two meshes of the two participants (p values can be found in the statistical reports available in the data repository or in the summary table) but did not show a strong correlation with any relevant parameter of our setup (angle_orig_deg 0.410484679, angle_calc_deg 0.410484558, $\vec{t}_{e}$ 0.013278187, $\vec{d}_{axis}$ 0.23777786) and just like in the case of the rotation angle, the difference is so small (for all of the angle groups the absolute mean and the standard deviation values are in the 10^-7 scale or less, calculated in mm) that also can be considered to be negligible. The most crucial registered values, the displacement of the axis ($\vec{d}_{axis}$; the distance of the original axis and the calculated ICR) and the EcD_ratio, were not mesh dependent because these results provided a statistically insignificant difference between the two meshes of the two participants (p values can be found in the statistical reports available in the data repository or in the summary table).

**References**

1. Eberharter JK, Ravani B. Kinematic registration in 3D using the 2D Reuleaux method. Journal of Mechanical Design, Transactions of the ASME. 2006;128: 349–355. doi:10.1115/1.2159027

| Wilcoxon Signed Rank Test Results (p value) | | | | | | | | | | | | |
| --- | --- | --- | --- | --- | --- | --- | --- | --- | --- | --- | --- | --- |
|  | columns | angle_calc vs angle_orig | | angle_calc between meshes | angle_important (measured) vs angle_important_should_be | | angle_important between meshes | rms against 0 | | rms between meshes | d_axis | EcD_Ratio |
|  | mesh | 1 | 2 | 1 vs 2 | 1 | 2 | 1 vs 2 | 1 | 2 | 1 vs 2 | 1 vs 2 | 1 vs 2 |
| rotation degree | 2 | 0.124 | 0.854 | 0.129 | 0.674 | 0.448 | 0.466 | 0.000 | 0.000 | 0.778 | 0.564 | 0.253 |
|  | 2.5 | 0.003 | 0.512 | 0.002 | 0.182 | 0.083 | 0.315 | 0.000 | 0.000 | 0.182 | 1.000 | 0.063 |
|  | 3 | 0.000 | 0.000 | 0.001 | 0.182 | 0.473 | 0.845 | 0.000 | 0.000 | 0.667 | 0.317 | 0.685 |
|  | 3.5 | 0.013 | 0.364 | 0.000 | 0.215 | 0.099 | 0.630 | 0.000 | 0.000 | 0.000 | 1.000 | 0.326 |
|  | 4 | 0.000 | 0.013 | 0.000 | 0.525 | 0.186 | 0.790 | 0.000 | 0.000 | 0.000 | 1.000 | 0.479 |
|  | 4.5 | 0.157 | 0.181 | 0.000 | 0.353 | 0.335 | 0.652 | 0.000 | 0.000 | 0.000 | 1.000 | 0.307 |
|  | 6 | 0.566 | 0.870 | 0.279 | 0.073 | 0.886 | 0.704 | 0.000 | 0.000 | 0.000 | 1.000 | 0.786 |
|  | 9 | 0.663 | 0.000 | 0.000 | 0.594 | 0.189 | 0.845 | 0.000 | 0.000 | 0.000 | 0.317 | 0.388 |
|  |  |  |  |  |  |  |  |  |  |  |  |  |
|  | no significant difference (p >= 0.05) | | | |  |  |  |  |  |  |  |  |
|  | there is siggnificant difference (p < 0.05) | | | |  |  |  |  |  |  |  |  |

**angle_calc:** The rotation angle of the calculated transformation

**angle_orig:** The rotation angle of the original transformation

**angle_important:** The described 90 - (alpha/2), using the calculated rotation angle as alpha

**angle_important_should_be:** The described 90 - (alpha/2), using the original rotation angle as alpha

**rms against 0:** The calculated rms value against 0.0 value (One-Sample Wilcoxon Signed Rank Test)

**rms between meshes:** Investigation whether the two dataset belonging to the two meshes generate significantly different rms values or not?

**d_axis:** Difference between the original axis and the calculated axis (avg at end points), check that is there any significant differences between these values of the different meshes

**EcD_Ratio:** A ratio that could be interpreted as 1mm translation error on the level of the mesh, causing {EcD_Ratio}mm translation error of the registered rotation axes
