# Supplementary material for: Instantaneous center of rotation, the first step to build up the digital laboratory of complex motions
Source: PLoS One. 2025 Aug 7;20(8):e0329021. doi: 10.1371/journal.pone.0329021 (PMC12331127; doi:10.1371/journal.pone.0329021)
Supplement: S2 Text — (DOCX) [file pone.0329021.s006.docx]

**The detailed presentation of these results and the related statistics of the second experimental setup: Effects of the registration errors of the axes of the positions of the rotated objects**

Note: A summary table of the *p* values are attached to the end of this document, statistical analysis reports are also available in the data repository

We found higher mean Axis error-caused Final error (AEcFE) values on the parallel groups, which gradually increases as they move further from the gravity points of the meshes. Thus, the same amount of error has less effect on the mesh position from a further direction. The perpendicular and circular error-type intergroup results corroborate this finding (e.g., Group G has higher individual and cumulative values than the similarly modified but further positioned FG Group). An in-depth investigation of the results at the subset level shows a similar pattern. The rms_after values of the parallel shifted error type were lower on those meshes belonging to the axes placed closer to the gravity point than those belonging to the further positioned axes, both modified by the same amount of error only in the opposite direction. Detailed information can be found in the S2 Table column *-direction: +direction rms_value ratio*. The ratio is never 1:1 in cases of the parallel error type.

The perpendicular error-type mean AEcFE values of the same group are much smaller than the parallel type with the same amount of error; thus, this direction is more sensitive to the error. However, these AEcFE values remain stable in the intergroup statistics, even if the central axes are distributed on a parallel path. The mean AEcFE values of the perpendicular error type decrease slightly on the parallel path, whereas the circular error-type changes only to a negligible extent. More precisely, the mean AEcFE parallel values increased by 49.43%–50.43% for Mash I and 49.19%–50.05% for Mesh II from Group A to Group F on the parallel Group path. The perpendicular value decreased slightly by less than 1%, and the circular value was only on the 0.01% scale. All these mean AEcFE values remain stable in the intergroup comparison on the perpendicular path, with the highest change in the parallel error type (1.4%–2%), slightly smaller on the further path originating from Group F. There is no change in these values on the circular path between the groups. We could interpret these findings as circular zones around the meshes which produce similar sensitivity to the registration errors. Parallel errors have the smallest effect on the mesh position, gradually decreasing further from the mesh, whereas errors in the perpendicular direction are more clinically dangerous.

Despite the dataset being grouped by the rotation angles we have not found fundamental differences in the test results between these groups. We found significant difference between the rms_after values of the two meshes if all error types were treated as single-grouped data and divided by the error types for all angle groups. These differences can be explained as consequences of unique meshes properties (shape, complexity, resolution, number of points), as in case of this Setup the performed transformations do not substitute the original rotation (contrary to the case of the First experimental setup), and the rms error values are calculated using the vertex points of the meshes, thus basic properties like the number of points in the meshes, have fundamental effects on the analysis. Thus, in the case of failed axis registrations, our findings can be interpreted as general trends characterizing the effects of unsuccessful registrations rather than strict numerical conclusions, contrary to the findings regarding ICR registrations where mesh properties do not change the registration results similarly.

As expected, the perpendicular and circular error types produced significantly different rms_after values (in all groups) compared to the parallel errors for both meshes. The difference between the perpendicular and circular groups was much smaller but still comprehensible by reviewing the derived values such as AEcFE. However, the rms_after values of these two groups proved statistically insignificant (except for the -1° groups). A cautious interpretation of these results is advised because the fundamental differences between the two error types seem to be clear contextually.

Detailed examination of the results shown in the S2 Table might be time-consuming. Furthermore, numerical differences in the results are granted when changing the observed body because various mesh properties partially modify the results. For easier interpretation of these numerical findings, an oversimplified summary is advised for the authors of this paper. The distance of the original axes from the gravity points of the meshes varied between 56 mm and 86 mm. In this range, the parallel error-type mean AEcFE ratios changed from 79.6 ± 15.7 to 118.8 ± 15.9 in the 3° group, from 119.7 ± 23.6 to 179.6 ± 24.0 in the 2° group, and from 239.8 ± 47.3 to 360.8 ± 48.2 in the 1° group for Mesh I. The analog values for Mesh II were 74.4 ± 14.5–111.0 ± 14.7; 111.9 ± 21.9–167.5 ± 22.3, and 224.1 ± 43.8–336.3 ± 44.7, respectively.

In the circular error type, the mean AEcFE values were 19.2 ± 0.05 in the 3° group, 28.7 ± 0.07 in the 2° group, and 57.4 ± 0.1 in the 1° group, without mentionable change between the meshes and different locations. The interpretation of these data shows that the least-tolerant AEcFE value of the circular error type (19.2 mean value) indicates that an approximately 2 mm axis registration error would exceed the clinically acceptable 0.1 mm error level, whereas parallel errors would cause at least four times less error on the level of the rotated scans [1]. The system might tolerate errors as great as 3–4 cm in some settings. These experimental results are hardly applicable in clinical situations, yet they describe some fundamental properties of the system. These findings might help structure clinical trials on the topic in the future.

**References**

1. Piehslinger E, Bauer W, Schmiedmayer HB. Computer simulation of occlusal discrepancies resulting from different mounting techniques. J Prosthet Dent. 1995;74: 279–283. doi:10.1016/S0022-3913(05)80135-0

| Test Results (p value) | | | | | | | | | | | |
| --- | --- | --- | --- | --- | --- | --- | --- | --- | --- | --- | --- |
|  | | Releated-Samples Wilcoxon Signed Rank Test | | | | Independent-Samples Kruskal-Wallis Test | | | | | |
|  | columns | rms all(circular, paralel, perpendicular) | rms circular | rms paralel | rms perpendicular | rms circular against rms perpendicular | | rms circular against rms paralel | | rms paralel against rms perpendicular | |
|  | mesh | 1 vs 2 | 1 vs 2 | 1 vs 2 | 1 vs 2 | 1 | 2 | 1 | 2 | 1 | 2 |
| rotation degree | -3 | 0.000 | 0.000 | 0.000 | 0.000 | 0.659 | 0.602 | 0.000 | 0.000 | 0.000 | 0.000 |
|  | -2 | 0.000 | 0.000 | 0.000 | 0.000 | 0.672 | 0.616 | 0.000 | 0.000 | 0.000 | 0.000 |
|  | -1 | 0.000 | 0.000 | 0.000 | 0.000 | 0.717 | 0.661 | 0.000 | 0.000 | 0.000 | 0.000 |
|  | 1 | 0.000 | 0.000 | 0.000 | 0.000 | 0.717 | 0.661 | 0.000 | 0.000 | 0.000 | 0.000 |
|  | 2 | 0.000 | 0.000 | 0.000 | 0.000 | 0.672 | 0.616 | 0.000 | 0.000 | 0.000 | 0.000 |
|  | 3 | 0.000 | 0.000 | 0.000 | 0.000 | 0.659 | 0.602 | 0.000 | 0.000 | 0.000 | 0.000 |
|  |  |  |  |  |  |  |  |  |  |  |  |
|  |  |  |  |  |  |  |  |  |  |  |  |
|  | no significant difference ( p >= 0.05) | | | |  |  |  |  |  |  |  |
|  | there is siggnificant difference (p < 0.05) | | | |  |  |  |  |  |  |  |
