# Supplementary material for: Instantaneous center of rotation, the first step to build up the digital laboratory of complex motions
Source: PLoS One. 2025 Aug 7;20(8):e0329021. doi: 10.1371/journal.pone.0329021 (PMC12331127; doi:10.1371/journal.pone.0329021)
Supplement: S3 Text — (DOCX) [file pone.0329021.s007.docx]

**Statement of novelty and** **relation of the presented mathematical background to previous publications.**

We examined the fundamental logical background behind the ICR theory. Most of our findings are geometrically predefined and are in agreement with previous publications [1]. Although Chen presented the clinical limitations of ICR calculations, it remains an essential investigative tool because it is the most profound approach to managing complex joint motions [25]. Mehl established a solid ground for understanding error sensitivity based on his fundamental kinetics. Although we used a different mathematical approach, it is crucial to state that it is merely an alternative to the previously published derivation of Mehl’s [2]. His solution is based on a continuous manner of the problem; therefore, his final derivation uses a few assumptions (Equations 17, 21, and 22 were reserved for small rotation angles, <15°, as reductions were made; furthermore, approximations were necessary to address his more complex kinematic model). Our simplified geometrical approach, which manages the problem as a discrete two-position system, provided an exact and comprehensible formula with a definite outcome, free of predefined assumptions and limitations in the rotation angles if the axes were perpendicular to the translational vector. For clarity, it is critical to state that our polar coordinate variation is convertible to Mehl’s Cartesian prescription and conclude similarly if *∆t* = 1; thus, we cannot state novelty in that regard. Putting it in context, the kinematic method can be simplified if the number of stages is limited to two.

Furthermore, some publications presented mathematical solutions for the explicit determination of the position of the ICR by *x*–*y* coordinates [3–5]. Thus, while these formulations can be applied as alternatives to the Reuleaux method to find ICRs, we found that an alternative geometrical approach would be advantageous because it handles the translation component as a variable input parameter of the complex motion.

**References**

1. Pogáts F. Egybevágóságok, gráfok, felületek. Budapest: ELTE TFK; 1993.

2. Mehl A. Is it possible to detect a true rotation axis of the temporomandibular joint with common pantographic methods? A fundamental kinematic analysis. Comput Methods Biomech Biomed Engin. 2020;23: 445–455. doi:10.1080/10255842.2020.1724975

3. Chen X. The instantaneous center of rotation during human jaw opening and its significance  in interpreting the functional meaning of condylar translation. Am J Phys Anthropol. 1998;106: 35–46. doi:10.1002/(SICI)1096-8644(199805)106:1<35::AID-AJPA3>3.0.CO;2-C

4. Crisco JJ, Chen X, Panjabi MM, Wolfe SW. Optimal marker placement for calculating the instantaneous center of rotation. J Biomech. 1994;27: 1183–1187. doi:10.1016/0021-9290(94)90059-0

5. Spiegelman JJ, Woo SLY. A rigid-body method for finding centers of rotation and angular displacements of planar joint motion. J Biomech. 1987;20: 715–721. doi:10.1016/0021-9290(87)90037-6
