# Supplementary material for: Instantaneous center of rotation, the first step to build up the digital laboratory of complex motions
Source: PLoS One. 2025 Aug 7;20(8):e0329021. doi: 10.1371/journal.pone.0329021 (PMC12331127; doi:10.1371/journal.pone.0329021)
Supplement: S4 Text — (DOCX) [file pone.0329021.s008.docx]

**The detailed discussion of the issue of error sensitivity on the clinical tools of digital dentistry.**

We presented our results that highlight the error sensitivity of the ICR system. We must determine whether these errors act in the same way with clinical tools designed to reproduce patient-specific motion. Do they compromise the outcomes of different clinical workflows based on virtual articulators and motion-tracking devices? All these tools are exposed to the mentioned vulnerabilities at least once in the workflow. Digital impression techniques are subjects of rapid evolution [1]. However, some concerning results have been reported on bite alignment accuracy [2–5], similarly to our previous findings [6]. If these devices repeatedly fail to meet clinically acceptable norms (maximum error level below 0.1 mm) defined by the scientific community [3,7], it would reveal the weakness of different quality assurance regulations for medical devices. These tools are available in the market without built-in standardized digital features that provide transparency on bite alignment accuracy, ensuring that they meet the standards and requirements established by evidence-based medicine. Performing such inquiries on exported raw data is challenging for some products and results in various incomparable protocols published in the literature, struggling to fulfill neglected institutional patient safety responsibilities. The artificial transformations of different digital toolsets might further magnify the errors of such poor performance.

We provided detailed discussions of the basic characteristics of the system, also an intellectual debt we now aim to fulfill because recent clinical studies have been published, citing our clinical results, which we then failed to discuss thoroughly, lacking the conclusions of this presented article [6,8,9]. Mouchoux et al.’s studies on landmark identification for analysis of the temporomandibular joint in real-time MRI. They aimed to improve the most critical element of these models, the accurate capture of the observed body. These systems proved to be highly sensitive for registration errors. The registration of the exact position of the observed body is fundamental because the smallest error easily overshadows any naturally present translational component of the biological (or other) system. Impaired registration of anatomical boundaries on MRI recording, segmentation errors of bones, and flawed bite alignment of intraoral scans gravely compromise the experimental results.

**References**

1. Eggmann F, Blatz MB. Recent Advances in Intraoral Scanners. J Dent Res. 2024. doi:10.1177/00220345241271937

2. Zheng Y, Zhang T, Liu Y, Pradies G, Wang X, Ma D, et al. Evaluation Methods and Influencing Factors on Accuracy for Static Virtual Articulation Obtained by Intraoral Scanners: A Critical Review of the Literature. J Esthet Restor Dent. 2024. doi:10.1111/jerd.13312

3. Rutkūnas V, Jegelevičius D, Gedrimienė A, Auškalnis L, Eyüboğlu TF, Özcan M, et al. Effect of Different Intraoral Scanners on the Accuracy of Bite Registration in Edentulous Maxillary and Mandibular Arches. J Dent. 2024;146. doi:10.1016/j.jdent.2024.105050

4. Kakali L, Halazonetis DJ. A novel method for testing accuracy of bite registration using intraoral scanners. Korean J Orthod. 2023;53: 254–263. doi:10.4041/kjod22.199

5. Revilla-León M, Gomez-Polo M, Zeitler J, Barmak AB, Kois J, Alonso J. Does the available interocclusal space influence the accuracy of the maxillomandibular relationship captured with an intraoral scanner? J Prosthet Dent. 2022;132. doi:10.1016/j.prosdent.2022.09.004

6. Safrany-Fark A, Laczi B, Nagy A, Lengyel L, Piffko J, Segatto E. A novel approach for determining instantaneous centers of rotation of the mandible with an intraoral scanner: A preliminary study. PLoS One. 2023;18: 1–15. doi:10.1371/journal.pone.0285162

7. Piehslinger E, Bauer W, Schmiedmayer HB. Computer simulation of occlusal discrepancies resulting from different mounting techniques. J Prosthet Dent. 1995;74: 279–283. doi:10.1016/S0022-3913(05)80135-0

8. Mouchoux J, Sojka F, Kauffmann P, Dechent P, Meyer-Marcotty P, Quast A. An automatic tracking method to measure the mandibula movement during real time MRI. Sci Rep. 2024;14: 24125. doi:10.1038/s41598-024-74285-9

9. Mouchoux J, Meyer-Marcotty P, Sojka F, Dechent P, Klenke D, Wiechens B, et al. Reliability of landmark identification for analysis of the temporomandibular joint in real-time MRI. Head Face Med. 2024;20. doi:10.1186/s13005-024-00411-7
