# Supplementary material for: Instantaneous center of rotation, the first step to build up the digital laboratory of complex motions
Source: PLoS One. 2025 Aug 7;20(8):e0329021. doi: 10.1371/journal.pone.0329021 (PMC12331127; doi:10.1371/journal.pone.0329021)
Supplement: S1 File — The setup was built up by two points rotated by the same amount on different circular paths because they would be two points of the same rigid body. After the rotation, both points shifted on the parallel lines by the same amount, representing the translational component. The Reuleaux method (purple lines in the presented S1 File GeoGebra Project) can be used to find the ICR. After manipulating the original values, the correctly constructed set maintains equal translation and rotation values. Our findings can be empirically verified using this straightforward tool. (ZIP) [file pone.0329021.s003.zip › S1 GeoGebra Project/S1 GeoGebra Project/mvxuqqabz-SI-Material.zip!/GeoGebra/HTML5/5.0/web3d/html/windowslive.html]

Callback for windows live service
